# Self-reported cognitive dysfunction and memory impairment in Systemic Autoimmune Rheumatic Diseases (SARDs): a mixed methods analysis of the INSPIRE cohort

**DOI:** 10.1093/rheumatology/keaf429

**Published:** 2025-08-07

**Authors:** Avni Varshney, Thomas A Pollak, Arjoon Arunasalam, Efthalia Massou, Felix Naughton, James A Bourgeois, Lucy Calderwood, Guy Leschziner, Alessandra Bortoluzzi, Alice Tunks, Ellie Dalby, Martha Piper, Laura Andreoli, Xiaofeng Yan, Sharmilee Gnanapavan, Arvind Kaul, David D’Cruz, Melanie Sloan

**Affiliations:** Institute of Psychiatry, Psychology and Neuroscience, King’s College London, South London and Maudsley NHS Foundation Trust, London, UK; Institute of Psychiatry, Psychology and Neuroscience, King’s College London, South London and Maudsley NHS Foundation Trust, London, UK; Institute of Psychiatry, Psychology and Neuroscience, King’s College London, South London and Maudsley NHS Foundation Trust, London, UK; Department of Public Health and Primary Care Unit, University of Cambridge, Cambridge, UK; Faculty of Medicine and Health Sciences, University of East Anglia, Norwich, England, UK; Department of Psychiatry and Behavioral Sciences, University of California, Davis Medical Center, Sacramento, CA, USA; Patient and Public Partners; Department of Neurology, Guy’s and St Thomas’ Hospitals NHS Foundation Trust, London, UK; Rheumatology Unit, Department of Medical Sciences, University of Ferrara and Azienda Ospedaliero-Universitaria S. Anna, Ferrara, Italy; Institute of Psychiatry, Psychology and Neuroscience, King’s College London, South London and Maudsley NHS Foundation Trust, London, UK; Patient and Public Partners; Institute of Psychiatry, Psychology and Neuroscience, King’s College London, South London and Maudsley NHS Foundation Trust, London, UK; Unit of Rheumatology and Clinical Immunology, ASST Spedali Civili, Department of Clinical and Experimental Sciences, University of Brescia, Brescia, Italy; Department of Psychiatry and Behavioral Sciences, University of California, Davis Medical Center, Sacramento, CA, USA; Department of Neurology, Bart’s Hospital, London, UK; Department of Rheumatology, St George’s Hospital, London, UK; The Louise Coote Lupus Unit, Guy’s and St Thomas’ Hospitals NHS Foundation Trust, London, UK; Department of Public Health and Primary Care Unit, University of Cambridge, Cambridge, UK; Faculty of Medicine and Health Sciences, University of East Anglia, Norwich, England, UK

**Keywords:** systemic autoimmune rheumatic diseases, SARDs, cognitive dysfunction, mixed methods, participation in daily life, quality of life, mental health, memory impairment

## Abstract

**Objectives:**

To explore self-reported cognitive dysfunction, including memory impairment, across systemic autoimmune rheumatic diseases (SARDs) and examine its impact and associations with demographic, clinical and psychosocial factors.

**Methods:**

A mixed-methods approach was employed, surveying 1853 SARD patients and 463 controls using validated instruments including the Everyday Memory Questionnaire-Revised (EMQ-R). Kruskal–Wallis tests and Spearman’s rank correlations were used to compare the groups. Additionally, 67 in-depth interviews were conducted for qualitative thematic analysis.

**Results:**

Systemic lupus erythematosus (SLE), undifferentiated connective tissue disease (UCTD) and Sjögren’s patients reported significantly higher rates of memory impairments than other groups. There was no evidence of increased self-reported memory impairment with disease duration or age. Moderate positive associations were found between EMQ scores and the lifetime frequency of all other neuropsychiatric symptoms. EMQ-R was positively associated with self-assessment of overall disease activity (r = 0.291, *P* < 0.001) and negatively correlated with well-being (r = −0.397, R^2^ = 0.159). Expanding on the quantitative findings, qualitative analyses highlighted the adverse impact of cognitive dysfunction on daily participation in activities, social isolation, self-esteem and mental well-being, and the potential underreporting of these symptoms to clinicians.

**Conclusion:**

This study highlights a significant impairment of memory in SARDs, notably in SLE, UCTD and Sjögren’s, and the impact of cognitive impairment on daily lives and well-being. The positive associations with disease activity and neuropsychiatric symptoms, and negative association with well-being emphasizes the need for targeted interventions. Future research should prioritize developing pharmacological and psychosocial interventions to address cognitive dysfunction in SARD patients. While reassuringly there was no evidence of worsening memory impairment over time, the underreporting of symptoms also suggests that cognitive issues may be more prevalent than clinical records indicate and thus emphasize the importance of thorough patient assessment.

Rheumatology key messages

- Self-reported memory impairment was highest in SLE, UCTD and Sjögren’s, and lowest in PMR.
- Cognitive dysfunction severely impacts many SARD patients’ lives and requires targeted support.
- There was no positive association between self-reported memory impairment and time since diagnosis.

## Introduction

Systemic autoimmune rheumatic diseases (SARDs) are a group of diseases where the immune system becomes overly aggressive and directed against the self, attacking the body’s tissues. This leads to diverse symptoms, often involving joints, muscles and multiple organs [1]. SARDs include connective tissue diseases—such as systemic lupus erythematosus (SLE), systemic sclerosis (SSc), inflammatory myopathies, Sjögren’s, and both undifferentiated and mixed connective tissue diseases (UCTD and MCTD)—as well as other inflammatory and immune-mediated conditions, including inflammatory arthritis (IA) such as rheumatoid arthritis (RA), systemic vasculitis and polymyalgia rheumatica (PMR). An increasingly recognized characteristic of SARDs is neuropsychiatric symptoms, linked to heightened morbidity, mortality and diminished quality of life [2]. Aside from neuropsychiatric lupus (NPSLE) [3], investigations into neuropsychiatric symptom domains across SARDs are scarce, primarily focusing on depression and anxiety [4].

Cognitive dysfunction, characterized by deficits in cognitive abilities such as memory, attention, processing speed and executive function, remains an under-investigated neuropsychiatric symptom in this population. Research highlights the presence of cognitive dysfunction in SARDs [5–9]. It can have a substantial negative impact on patients, with lost work productivity causing a substantial economic burden [10], greater functional disability and poorer quality of life [11]. Memory impairment is a specific symptom that has also been shown to affect quality of life severely [12] and warrants further investigation. Additionally, the predominant focus on objective measures of cognitive dysfunction [2, 5–9, 11, 13] in current literature largely overlooks the lived experience, functional impairment and psychosocial impact of these symptoms. Furthermore, Meade *et al.* [6] through their systematic review observed inconsistency in the association among cognitive dysfunction and demographic, clinical and psychological factors across various studies.

Thus, our research aims to bridge these gaps by investigating self-reported cognitive dysfunction both qualitatively and quantitatively, with a focus on memory impairment. We investigate their association with demographics, disease groups, disease duration, other SARD symptoms and quality of life. This research aspires to contribute to a holistic understanding of SARDs, transcending specific disorders.

## Methods

### Study design

This study is part of the INSPIRE (**I**nvestigating **N**europsychiatric **S**ymptom **P**revalence and **I**mpact in **R**heumatology Patient **E**xperiences) research project [14]. The project employs a mixed-method approach utilizing cross-sectional surveys and in-depth interviews. This research was conducted with a patient-centred approach, evidenced by the meaningful and equal involvement of patient partners in the research team.

The survey was designed using a rigorous, collaborative approach with input from patients, clinicians and rheumatology charities. Pre-survey interviews explored broader SARD-related topics, including neuropsychiatric symptoms, patient experiences and societal issues like trust and stigma.

Pilot surveys and social media discussions guided the integration of a wide range of neuropsychiatric symptoms, prioritizing patient concerns over existing literature. Medical professionals reviewed symptom phrasing, which was finalized in consultation with patients.

To manage the survey’s length and complexity, participants could pause and resume to reduce fatigue. Symptom ordering was randomized to minimize bias. The survey was made available globally, aiming to gather comprehensive data for the INSPIRE research project.

### Measures

The INSPIRE survey included questions on demographics, diagnostic history, symptom experiences, medication, healthcare encounters and open-ended questions on the impact of symptoms on daily life. Symptom experiences were assessed using a 5-point Likert scale, with each symptom listed alongside descriptions developed through patient-clinician collaboration. The survey also included validated tools to assess participants’ anxiety (through GAD-7) [15], depressed mood (through PROMIS depression short-form) [16] and well-being (through Warwick-Edinburgh Mental Well-being Scale, WEMWBS) [17].

The key instrument used was the Everyday Memory Questionnaire–revised (EMQ-R) [18], a 13-item self-report measure assessing everyday memory difficulties. It employs a 5-point Likert scale, with higher scores indicating greater perceived memory difficulties. The EMQ-R has strong psychometric properties, including internal reliability and discriminant validity. Its utility has been established in various healthy and neurological conditions [18–20].

The survey also assessed current subjective levels of pain, fatigue and measures of adaptation to life with a chronic disease (ADAPT) using one-item questions with a scale of 0–100. ADAPT questions were crafted in collaboration with patients and clinicians to understand patients’ experiences of living with SARD diseases. The questions included “How do you feel overall in terms of being satisfied with your life,” “How have you adapted to the changes in your life from having a chronic disease,” “How much do you participate in everyday life,” and “How much control do you feel you have over your disease symptoms.”

Additionally, interviews were conducted to acquire qualitative data. These semi-structured interviews commenced with pivotal open-ended inquiries, with subsequent questions guided by each participant’s experiences and priorities, aimed at uncovering and exploring diverse neuropsychiatric symptoms, their medical experiences with healthcare providers, their level of trust, mental health and well-being, and understanding their concerns and perspectives relevant to the project.

### Procedure

Prospective patient participants were recruited internationally through online forums, social media groups and international SARD charities. They were invited to access the information sheet, consent form and survey hosted on Qualtrics. Upon completing the survey, the concluding page included a request for participants to forward the link to a control participant version of the survey to a friend. Purposive sampling was used to contact patients who agreed to be interviewed by a trained medical researcher. Interviews mostly took place over Zoom, with a minority being conducted in person, over the telephone or via email. Informed consent was obtained electronically at the start of the surveys and re-recorded verbally for interviews.

### Analysis

SPSS Software V29 was used to analyse the quantitative data, with the significance level set at *P* < 0.05. The Kruskal–Wallis and Mann–Whitney U tests assessed differences across groups for EMQ-R results. The statistical software automatically applied Bonferroni corrections to control for Type 1 errors (false positives). Spearman’s correlation was used to assess the association between EMQ-R scores and time since diagnosis, neuropsychiatric symptoms, depressed mood, anxiety, well-being, pain, fatigue and quality of life domains. Our co-designed ADAPT measures included: participation in daily activities, adaptation to the disease, feeling control over their lives and satisfaction with life. For qualitative data, thematic analysis was employed [21], utilizing NVivo14 software for data management and coding. Our larger multidisciplinary team validated the codes and themes, enhancing their reliability.

### Ethics and regulatory approvals

Ethical approval was obtained through the Cambridge Psychology Research Ethics Committee: PRE.2022.027. The INSPIRE study was pre-registered (https://osf.io/zrehm). This paper’s lead author (A.V.) completed a statistical analysis plan for approval by the study supervisors (M.S. and T.P.) before accessing any data.

## Results

Our analysis included 1853 SARD patients and 463 controls (Table 1). The patient cohort predominantly comprised White (93%) and UK residents (85%), who were female (91%). SLE (31%) and IA (25%) were the most frequented diagnoses. Excerpts explicitly referencing cognitive symptoms or the colloquial term “brain fog” were extracted from all interview transcripts (*n* = 67) and open-ended survey responses (*n* = 999).

**Table 1.** Participants’ sociodemographic and clinical characteristics

Characteristics	Patient survey (*n* = 1853)	Patient interviews (*n* = 67)	Control survey (*n* = 463)
**Age**			
18–30	94 (5%)	6 (9%)	45 (10%)
30–39	195 (11%)	5 (7%)	71 (15%)
40–49	298 (16%)	17 (25%)	82 (18%)
50–59	519 (28%)	16 (24%)	84 (18%)
60–69	478 (26%)	9 (13%)	120 (26%)
70+	267 (14%)	14 (21%)	60 (13%)
Prefer not to say	2 (<1%)	0 (0%)	1 (<1%)
**Gender**			
Female	1687 (91%)	60 (90%)	334 (72%)
Male	160 (9%)	7 (10%)	126 (27%)
Other/undisclosed	6 (<1%)	0 (0%)	3 (<1%)
**Country/region**			
England	1285 (69%)	38 (57%)	341 (74%)
Scotland	144 (8%)	7 (10%)	43 (9%)
Wales	104 (6%)	7 (10%)	20 (4%)
North Ireland	35 (2%)	3 (4%)	7 (2%)
USA or Canada	112 (6%)	4 (6%)	16 (3%)
Europe	121 (7%)	4 (6%)	24 (5%)
Asia	18 (1%)	1 (1%)	1 (<1%)
Latin America	4 (<1%)	0 (0%)	2 (<1%)
Australia or New Zealand	19 (1%)	2 (3%)	0 (0%)
Other	11 (<1%)	1 (1%)	9 (2%)
**Ethnicity**			
White	1718 (93%)	56 (84%)	434 (95%)
Asian	49 (3%)	7 (10%)	6 (1%)
Black	23 (1%)	2 (3%)	4 (1%)
Mixed	40 (2%)	2 (3%)	11 (2%)
Other/undisclosed	23 (1%)	0 (0%)	8 (1%)
**Disease**			N/A
SLE	566 (31%)	25 (37%)	
Inflammatory arthritis	456 (25%)	9 (13%)	
Vasculitis	200 (11%)	3 (4%)	
Sjögren’s syndrome	150 (8%)	6 (9%)	
PMR	132 (7%)	7 (10%)	
UCTD	77 (4%)	9 (13%)	
Myositis	64 (4%)	3 (4%)	
Systemic sclerosis	63 (3%)	2 (3%)	
Mixed/multiple	145 (8%)	3 (4%)	

### Differences across different diseases and sociodemographic groups, based on EMQ-R

#### Disease groups

**Figure 1.**
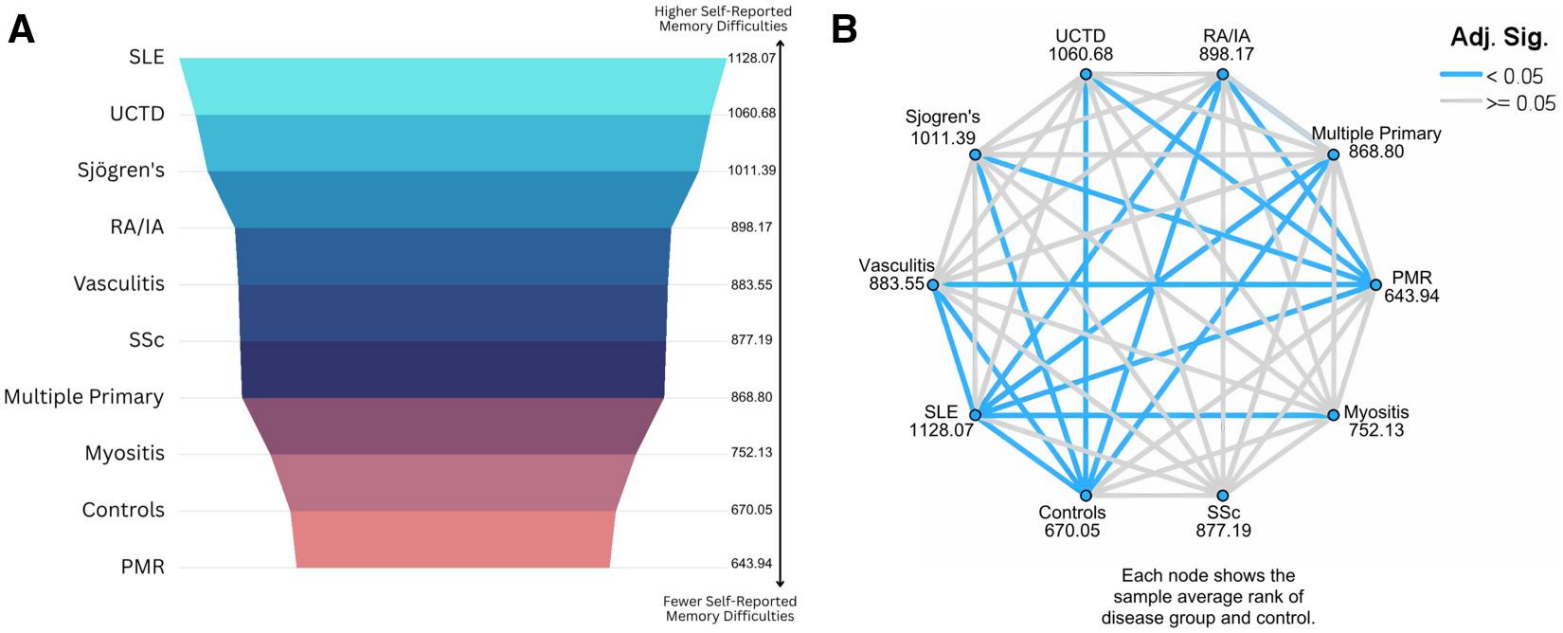
Disease comparisons of EMQ-R levels. (**A**) Ranking of SARD disease groups and controls based on memory difficulties. *Note*. Multiple primary refers to patients who had more than one primary diagnosis of SARDs. (**B**) Pairwise comparisons of disease and control EMQ-R scores

SARD groups were ranked based on the extent of impairment shown through EMQ-R scores (Fig. 1A), from highest dysfunction to lowest as (with median, interquartile range; Quartile 1—Quartile 3): **SLE** (median = 21, IQR = 23; 9.5–32.5) > **UCTD** (median = 18, IQR = 18.5; 8–26.5) > **Sjögren’s** (median = 16, IQR = 19; 7–26) > **RA/IA** (median = 12, IQR = 20; 5–25) > **Vasculitis** (median = 12, IQR = 19.75; 4–23.75) > **SSc** (median = 14, IQR = 16.75; 5–21.75) > **multiple primary** (median = 12, IQR = 18.75; 5–23.75) > **myositis** (median = 9.5, IQR = 15; 4–19) > **controls** (median = 7; IQR = 12; 3–15) > **PMR** (median = 7, IQR = 8; 4–12). Fig. 1B summarizes the statistical significance of pairwise differences in EMQ-R scores between disease groups. Everyday memory levels in myositis, PMR and SSc were not significantly different from controls.

#### Age

**Figure 2.**
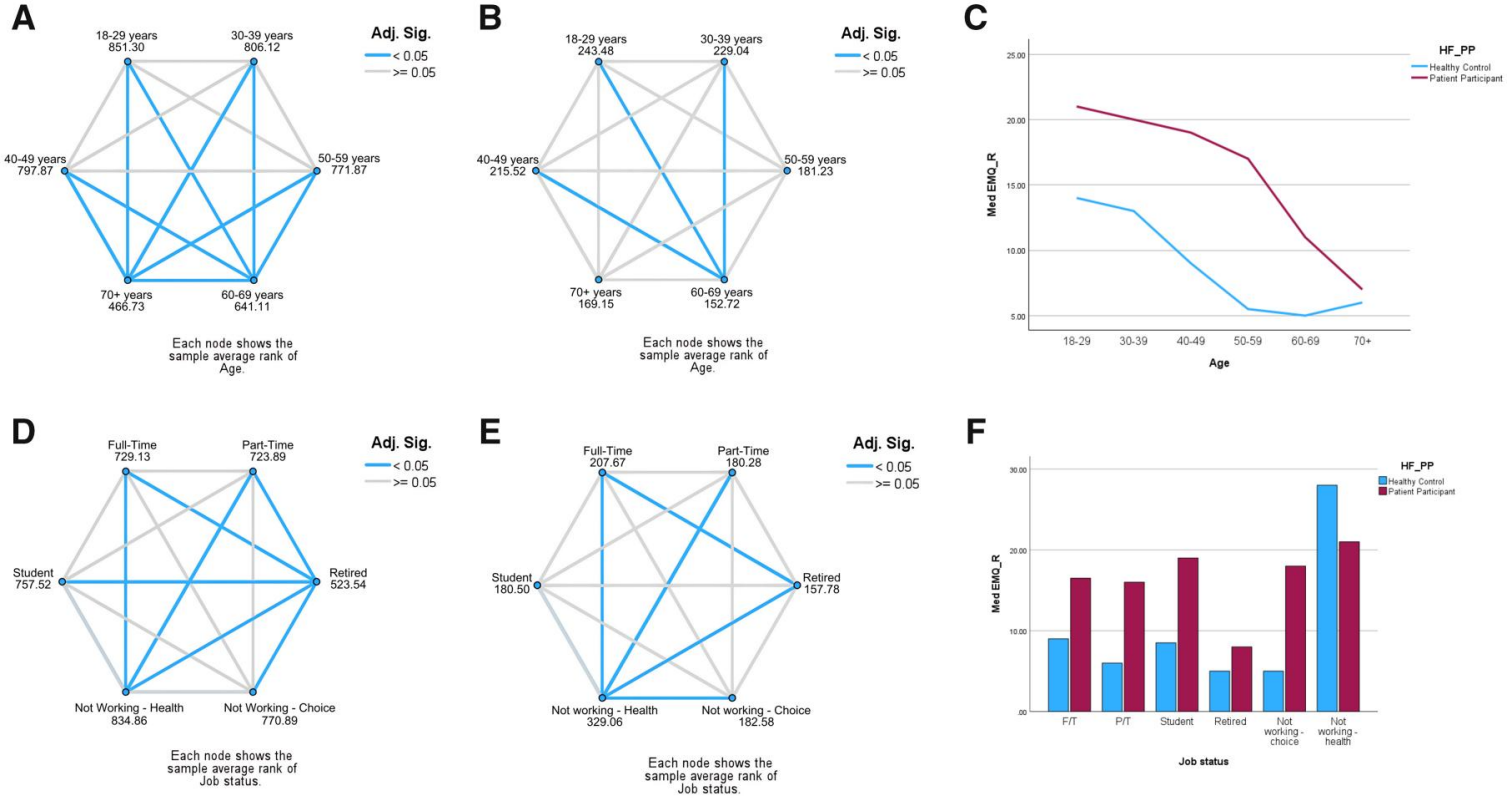
EMQ-R results for age groups and job status. (**A and B**) Pairwise comparison of age (**A**—patients; **B**—controls). (**C**) EMQ-R results across ages for patients and controls. (**D and E**) Pairwise comparison of job status (**D**—patients; **E**—controls). (**F**) EMQ-R results across job status for patients and controls

There was a weak negative correlation between age groups and EMQ-R scores for patients (−0.284, *P* < 0.001) and controls (−0.274, *P* < 0.001), with those aged 60+ reporting significantly *fewer* memory difficulties. Pairwise comparisons for patients are presented in Fig. 2A and for controls in Fig. 2B. Comparing patients to controls, significant differences were found in the 30–39, 40–49, 50–59 and 60–69 age groups (Fig. 2C).

#### Job status

Pairwise comparisons for patients and controls are shown in Fig. 2D and E, respectively. Among patients, individuals not working due to health reported the *highest* EMQ-R scores (with no significant difference with not working by choice or student status), while retirees reported the *least*. When comparing controls and patients, significant differences were observed for all except “not working due to health” and students.

#### Gender

Due to insufficient sample sizes of non-binary and other genders, only male and female data were analysed. Between patients, there were no significant gender differences in EMQ-R scores. Patients reported significantly higher EMQ-R scores than controls for both genders.

#### Ethnicity

No significant differences were noted in EMQ-R scores between Asian, Black, Mixed and White ethnicities. A significant difference was noted between participants and controls for Asian, White and Mixed groups.

### Correlation of EMQ-R scores with time since diagnosis

The only significant associations between EMQ-R scores and time since diagnosis were for UCTD (r = −0.287, *P* = 0.030) and SSc (r = −0.362, *P* = 0.011). These negative associations indicate that the longer the duration since the diagnosis, the lower the degree of self-reported memory impairment.

### Associations between memory and other SARD-related symptoms

Spearman’s correlation revealed that EMQ-R scores significantly correlated (*P* < 0.001) with all other neuropsychiatric symptoms measured (Table 2), with no notable differences between SARDs patients and controls.

**Table 2.** Correlation of EMQ-R scores with lifetime frequency of other measured SARD-related symptoms for patients and controls

SARD-related symptoms	Correlation (patients)	Correlation (controls)
Feelings of unreality	0.413	0.442
Restlessness/agitation	0.370	0.381
Disrupted dreaming sleep	0.367	0.297
Loss of coordination	0.363	0.322
OCD	0.355	0.387
Uncontrollable emotions	0.348	0.362
Headaches	0.345	0.224
Hypersensitivity	0.338	0.271
Negative sensory symptoms	0.328	0.326
Tremors	0.324	0.35
Positive sensory symptoms	0.320	0.34
Hallucinations	0.318	0.323
Disinhibition	0.311	0.29
Dizziness/raised heart rate on standing	0.311	0.241
Visual changes	0.305	0.277
Palpitations	0.284	0.301
Mania	0.281	0.332
Delusions or paranoia	0.275	0.262
Weakness	0.261	0.325
Hearing loss	0.252	0.198
Bowel/bladder dysfunction	0.239	0.183
Difficulty swallowing	0.230	0.271
Insomnia	0.215	0.209
Seizures/epilepsy	0.133	0.159
Tinnitus	0.111	0.161

*Note.* All the correlations are significant at the 0.01 level (two-tailed). All correlations between EMQ-R scores and SARD-related symptoms were significant at the *P* < 0.01 level, strongest associations being feelings of unreality, restlessness/agitation and disrupted dreaming/sleep.

### Association of EMQ-R scores with overall disease activity, depression, anxiety, pain and fatigue

There was a weak positive correlation between overall disease activity EMQ-R (r = 0.274, *P* < 0.001). Spearman’s correlation revealed significant associations between EMQ-R scores and all the other factors, with the strongest correlation with anxiety (r = 0.417, *P* < 0.001), followed by depression (r = 0.364, *P* < 0.001), fatigue (r = 0.314, *P* < 0.001) and pain (r = 0.285, *P* < 0.001).

### Association of EMQ-R scores with well-being and ADAPT measures

**Figure 3.**
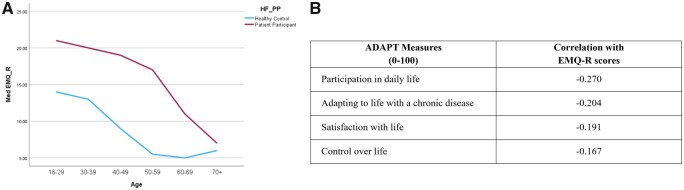
Associations of EMQ-R with well-being and ADAPT measures. (**A**) Correlation of EMQ-R with mental well-being (WEMWBS). (**B**) Correlations between EMQ-R with ADAPT measures. *Note*. All the correlations are significant at the 0.01 level

A negative correlation was observed between well-being (WEMWBS scores) and EMQ-R (r = −0.398, *P* < 0.001) (Fig. 3A), suggesting that greater memory impairment was associated with lower well-being. EMQ-R was also significantly negatively correlated with all the ADAPT measures (Fig. 3B).

### Thematic analysis of open-ended survey questions and interviews

The analysis of qualitative data (often colloquially referred to as “brain fog” by patients) provided a comprehensive understanding of the impact of cognitive dysfunction, including memory impairment, on patients’ lives, in the form of several themes:

### Theme 1: cognitive dysfunction reduces participation in all aspects of life, further impacting well-being

One of the prominent themes encompassed vastly reduced participation in daily life resulting from feelings of restriction due to cognitive dysfunction: “My life is exceptionally limited—particularly due to the brain fog and fatigue” (Ppt 1912, SLE). Adverse impacts of cognitive dysfunction were frequently reported as adversely impacting patients’ mental health by permeating all aspects of their lives, reducing participation in work/career: “I eventually gave up my much-loved career as I thought I was a liability with my memory problems” (Ppt 0679, SLE); social interactions: “I can’t always fully participate in conversations because of brain fog” (Ppt 0175, SLE); and daily activities, “I can’t shop on my own as I can’t remember what I'm getting” (Ppt 0052, UCTD).

The impact on work and career was particularly pronounced, with a striking majority of participants describing severe impairment and its adverse impacts on their self-esteem and well-being:

“The first symptom I felt after returning to work was the inability to concentrate and remember things, not being able to read and take in or remember what I'd read. Both of my (job) roles required me to be fully cognitive and have a Great memory. Because of this my self-esteem and confidence lessened and my mental health issues, which had been mild, started to deteriorate rapidly.” (Ppt 0701, Sjögren’s)

In addition to professional life, patients discussed how cognitive dysfunction adversely affected their participation in leisure activities:

“The cognitive effects, not being able to concentrate, so reading, a great love of mine, is very difficult. I have to reread pages and can only do a couple at a time, and it also gives me a headache—very distressing.” (Ppt 1474, vasculitis)

#### Sub-theme—self-isolation due to fear of being judged by others

Patients frequently reported feeling “stupid,” “embarrassed,” and “judged” (multiple ppts) due to cognitive dysfunction, which often led them to avoid socializing:

“The brain fog has changed everything, it’s not just what I can’t do anymore, it’s sort of like my social skills a bit now too, and my confidence…too embarrassed to even go out and see old friends sometimes.” (Ppt 0528, RA/IA)

Although many patients had restricted socializing of their own accord, this did not reduce the impact on mental health, and the subsequent feelings of isolation and loneliness: “don’t want to particularly want to be around people but feel desperately lonely” (Ppt 0043, UCTD).

### Theme 2—fears of progressive decline, although most relapse-remit and/or Plateau

Cognitive dysfunction was often described as fluctuating, frequently relapsing-remitting in line with other SARD symptoms. However, some patients discussed that, although there were fluctuations, they never returned to their pre-disease level of cognitive abilities, with many perceiving a permanent reduction in abilities. There was sometimes a fear that the initial impairment would be progressive with several stating that they thought they had dementia initially: “It was so bad, I thought it was, you know, Alzheimer’s, as my mum had that” (ppt 0495, SLE) or the symptoms were compared with dementia:

“I will start a sentence and forget the other half of the sentence after about three words…It’s very frustrating at times, as I am in my 20 s, the alleged prime of my life, and it makes me feel like a dementia sufferer…On bad days I wonder if this is as good as it gets. I am told that my condition is improving but have never made it to remission and it seems increasingly elusive.” (Ppt 0505, vasculitis)

It appeared that cognitive ability often plateaus. While not always returning to pre-disease levels, they did not appear to continue to deteriorate for the majority.

#### Sub-theme: comparison with pre-morbid ability

Many participants began discussions regarding their cognitive abilities by comparing them to their pre-morbid abilities, and how this reduction was often the most upsetting aspect:

“I knew all the kids’ names and then I didn’t remember the names of even one teacher…I just couldn’t cope…I was in charge of dinner money, and I’d have to reconcile it and I could never…it upsets me that I couldn’t do things (that) had been easy before.” (Ppt 0701, Sjögren’s)

Having to leave jobs due to this permanent reduction in cognition was common, with patients reporting substantial blows to their self-esteem and mental health, and describing feelings of professional failure and inadequacy:

“Being able to keep up with tasks at work and lots of brain fog and worries you may make a mistake. Needing time to relax after working. Not being able to keep up with demands and being felt as though you are failing.” (Ppt 1560, RA/IA)

### Theme 3: patient insight and instinct regarding attribution related to the severity and response of cognitive dysfunction

Although it was more common for patients with SLE, and UCTD to feel that their cognitive problems were wholly disease-related, other disease groups also mentioned that they considered the cognitive dysfunction to be due to direct mechanisms of effect:

“I noticed word acquisition when I was a public speaker became more difficult and memory of what I had said, a problem. Inflammation in the brain is real.” (Ppt 1267, RA/IA)

The symptom severity was also sometimes cited to explain participants’ reasoning that the dysfunction was less likely to be caused by the challenges of living with SARD and more likely the direct effect of the disease on the brain. Such severe cognitive dysfunction also raised concerns about physical well-being during flares. This included road safety, including two patients who detailed forgetting which colour traffic light meant it was safe to cross a road, and numerous patients reporting getting lost in familiar places:

“I couldn’t remember things…when I was having cerebral lupus because I was driving to my sisters and I just got lost, I couldn’t remember the way and I had to re-learn that route.” (Ppt 0132, SLE)

Although patients could lack insight at the time, in retrospect, these patients attributed their cognitive dysfunction entirely to the direct effects of the rheumatologic disease.

Various explanations were given for these attributional views including the positive response of the symptoms to corticosteroids: “…directly the disease because of how well it responds to steroids… and then the flare is confirmed via labs” (Ppt 0001, UCTD) and immunosuppression “I know they are due to Sjögren’s because medication has made a huge difference… Since re-starting medication, I have completed two degrees” (Ppt 1456, Sjögren’s).

Some participants discussed how their cognitive dysfunction was not reflected in their blood tests or other diagnostic test results, although a minority of patients cited objective tests as evidence of direct disease effect:

“My brain is altered, they could see that on the brain scan, more damage than a person of my age should have. I think my brain fog comes from that.” (Ppt 1511, SLE)

For those with less severe cognitive dysfunction, some patients perceived that anxiety could exacerbate or even cause cognitive difficulties: “With the anxiety, I also get that I can’t pay attention and concentrate, but that’s not related to the disease” (Ppt 0603, UCTD). Others described how the cognitive dysfunction was anxiety-provoking, rather than the converse: “I panic when I have word-finding problems on bad days, I feel foolish when I can’t find the most basic word, I get anxious to the point I feel I'm having a panic attack” (Ppt 0052, UCTD). These indicate a bidirectional relationship between anxiety and cognitive function, similar to the relationship between fatigue and cognitive function: “But if I'm very tired when I have fatigue, then I feel the brain fog” (Ppt 0978, SLE).

In addition to attributional views as to the direct or indirect effect of the disease, participants acknowledged the influence of ageing, particularly concerning memory lapses:

“I feel the brain fog, times when it is hard to think objectively, is part of Sjögren’s. But the general forgetfulness is also due to ageing.” (Ppt 0809, Sjögren’s)

### Theme 4: underreporting of symptoms to clinicians

The third theme identified revealed the impact of cognitive dysfunction on the way patients report their health and symptoms to their clinicians.

#### Sub-theme: underreporting of cognitive dysfunction to clinicians

Multiple participants mentioned that they had not reported their cognitive symptoms to their clinicians. A major reason was that patients felt that their physical symptoms, which were often considered to be more debilitating and visible, were more important to address. This belief led these patients—and their clinicians—to prioritize discussions about physical health over cognitive health, leaving cognitive dysfunction unaddressed. As one participant shared:

“It’s not something that is at the forefront like as much as the other symptoms are, like the pain and nausea, the muscle aches and the joint aches… I think you just forget about it. You forget to mention it as much… it’s just something that doesn’t come up as much. I don’t think they ever ask you anything about that.” (Ppt 1241, SLE)

#### Sub-theme: underreporting of other symptoms due to cognitive impairment

The impact of cognitive dysfunction extends beyond the underreporting of cognitive symptoms themselves. It can lead to incomplete reporting of *other* health issues due to forgetfulness, failure to relate symptoms, and/or feeling overwhelmed with managing the complex, simultaneous presentation of symptoms. This can result in a fragmented and incomplete clinical picture, affecting the overall management and treatment outcomes: “To the point, that not all of my most salient symptoms are ever mentioned due to memory issues” (Ppt 0730, SLE).

## Discussion

This study compared memory impairment in SARDs and its association with other symptoms and patient experiences. SLE, UCTD and Sjögren’s patients demonstrated the highest prevalence of self-reported everyday memory impairment (measured by the EMQ-R) relative to other SARDs, with PMR patients the least. Self-reported memory impairment was positively associated with all other neuropsychiatric symptoms and overall disease activity and negatively associated with mental well-being (measured by the WEMWBS) and all measures of life satisfaction. Qualitative analysis identified profound and multifaceted impacts of cognitive dysfunction on patients’ self-esteem, mental health and participation in daily life. Notably, no positive associations were found between memory impairment and age or disease duration. This finding provides evidence against progressive cognitive dysfunction, a common fear among patients, although a longitudinal study is required for more definitive evidence.

The disease-related comparisons align with previous research that also found significant differences in cognitive functioning (including memory impairment) between SLE and RA [6]; SLE and Sjögren’s [22]; RA and SSc [7]; vasculitis and RA [8]. The underlying pathophysiological mechanisms, including inflammation, immune dysregulation and vascular involvement, may contribute to the differential direct impact of these diseases on memory function [23]. Correlating EMQ-R scores with multiple neuropsychiatric and physical symptoms revealed significant positive associations for all symptoms. Unfortunately, this finding does not assist us in the difficult task of *attribution*, as many of these symptoms will also increase during a flare, along with cognitive dysfunction, including memory impairment.

Furthermore, depression, anxiety, pain and fatigue may impact cognitive processes by interfering with attention, memory and executive function [2, 24, 25]. Conversely, cognitive dysfunction may contribute to the development and maintenance of depressive and anxiety disorders through a cyclic process of negative affect and memory failure [26].

The level of direct attributability to the effect of the SARD on the brain will have a high degree of inter- and intra-person variation and requires careful consideration regarding whether immunosuppression will improve patient cognition and lives. Treatment of the underlying SARD has been reported to greatly improve cognitive function in some cases [27, 28], and reducing cognitive dysfunction by controlling systemic inflammation has been advocated in the literature [29]. Psychosocial support should also be considered for all affected patients due to the highly adverse impact identified on QoL.

While some of the more severe cognitive problems reported by our interviewees—such as getting lost in familiar places and being obviously confused—will be more readily identified and treated, clinicians must also consider the under-reporting of cognitive symptoms to clinicians. In accordance with Flythe *et al.* [30], our study participants also reported prioritizing discussing physical symptoms with clinicians and under-reporting cognitive and mental health symptoms. In addition to this prioritization, cognitive dysfunction and other neuropsychiatric symptoms were discussed as impairing a patient’s ability to recognize, remember or articulate their symptoms effectively.

The expectation of some deterioration in memory with ageing was at odds with our results demonstrating a negative association between age and EMQ-R scores for patients and controls, as also found in other studies using the EMQ [31]. This observation may be related more to the lower impact of, and concern about, memory difficulties among older people than an actual improvement in memory performance with ageing. Reasons may include more expectation and thus acceptance of some level of memory difficulties, age-related changes in memory perception [32], reporting, and factors related to employment status, such as stress levels [33]. Our findings of no evidence of progressive memory impairment with time since diagnosis is in line with longitudinal studies on cognitive dysfunction in SLE using objective measures [34].

Interestingly, findings suggested memory impairment in SSc and UCTD may decrease over time with the disease, contrasting with expected cognitive decline in other chronic conditions. Possible explanations include that patients might develop coping strategies and/or receive medications and interventions mitigating cognitive symptoms [35]. Additionally, initial diagnosis-related anxiety could temporarily worsen cognitive function, which could improve as patients adjust to their condition [36, 37]. However, the two disease groups with these findings had relatively low numbers of participants (<80), and the correlations were significant only at the 0.05 level and could represent Type 1 errors.

A negative correlation was found between memory difficulty measures and multiple measures of satisfaction and participation in life. These negative associations with well-being align with the literature [2, 13, 38–40]. Qualitative analysis, aligned with quantitative analysis, highlighted the profound and far-reaching impact of cognitive dysfunction on self-esteem and mental health by limiting the patients’ participation in activities [41–43]. The workplace was noticeably challenging, with cognitive dysfunction significantly impacting job performance and leading to unemployment or premature retirement in many cases. The loss of employment represented a financial setback [10] and a significant blow to self-identity, contributing to feelings of failure and inadequacy [44]. Beyond occupational challenges, participants reported frustration, distress and diminished life satisfaction due to limitations in leisure activities (like reading), as noted in the literature [45, 46]. Additionally, a reciprocal relationship was reported among social withdrawal, isolation and symptoms of anxiety and depression [47], potentially contributing to more cognitive dysfunction and accelerated decline. Qualitative data suggested that participation in everyday activities might mediate the relationship between cognitive dysfunction and satisfaction with life, which could be explored further.

The colloquial phrase “brain fog” was commonly used among patients. In the literature, the excessive and generalized use of this and similar terms (e.g. “fibro fog,” and “menopause brain fog” [48]) may generate an under-appreciation of the severity of some SARDs patients’ level of cognitive dysfunction. This can also include very severe dysfunction such as acute confusional state/delirium, as highlighted in the SLE criteria for neuropsychiatric lupus [49]. It is also important to recognize that what a clinician or society deems “mild dysfunction” may still have serious consequences for the patient, such as problems with study/employment, lowered self-esteem and social avoidance. Patients also rarely have a pre-disease cognitive assessment so later cognitive assessments may be deemed acceptable in terms of national averages yet be lower than the *specific* patient’s pre-disease cognitive function. The reported reduction in abilities was found to be particularly distressing.

This study has limitations, primarily including selection bias due to the self-selection of participants, potentially skewing the sample. Reliance on self-reported cognitive dysfunction may be influenced by mood, fatigue, and/or lack of insight, potentially leading to inaccurate reporting [50]. Unverified diagnoses, a cross-sectional design and the lack of objective cognitive measures limit the robustness of some findings. While self-reported and objective cognitive measures show differing associations with mood [51, 52], subjective impairments are arguably more relevant to patients’ quality of life. Notably, the sample was predominantly White and female (aged 40–60) potentially introducing demographic biases and limiting generalizability.

The study’s ethnic homogeneity (93% White) may be due to health literacy, research reluctance, or online recruitment biases, which prevents a meaningful analysis of cultural differences in cognitive experiences. The study team has now recruited an inequalities researcher and advisor to increase the engagement and trust in under-served communities. The findings of no differences between genders and ethnicities may relate to small subgroup samples, leading to type II errors. Furthermore, medication effects were not comprehensively controlled. Additionally, hormonal influences during perimenopause/menopause may also impact cognitive function, requiring more research, and examining the largely unexplored experiences of male patients would address important knowledge gaps.

Despite these limitations, this study offers several strengths that contribute to its significance in the field of cognitive dysfunction in SARDs. The large and diverse sample size, encompassing 1853 patients across multiple SARD conditions, and the co-designed nature of the INSPIRE study, provides extensive and novel data for analysis that is centred on patient priorities. The mixed methods approach comprehensively explores cognitive dysfunction, focusing on memory impairment, and offering statistical evidence and rich patient narratives. Additionally, the comprehensive symptom assessment, patient-centred approach, and the involvement of patient partners as equal and valued research team members increase the probability that the study captures issues most relevant to those living with SARDs. The study, serving its purpose as exploratory research, provides various avenues for subsequent, more detailed, studies.

## Conclusion

This study reveals significant differences in memory difficulties across SARDs, with SLE, UCTD and Sjögren’s patients reporting the most pronounced dysfunction. Importantly, we found that self-reported memory impairment was *not* positively associated with time since diagnosis or age, suggesting that memory impairment may not be progressive in the majority of SARDs cases. The study also illuminated the profound impact of cognitive impairment on patients’ self-esteem and mental health, with a bidirectional relationship between reduced participation in daily activities and social isolation. Therefore, effective management requires personalized approaches addressing psychosocial factors and optimal disease management. Longitudinal studies are needed to investigate within-person changes in cognitive function and attributability. Future research should focus on developing targeted interventions and improving awareness of the socially isolating impacts of cognitive dysfunction.

## Data Availability

Anonymized data will be available on reasonable request following the completion of the INSPIRE studies.
